# Areas of Law: Three Questions in Special Jurisprudence

**DOI:** 10.1093/ojls/gqac025

**Published:** 2022-10-26

**Authors:** Tarunabh Khaitan, Sandy Steel

**Affiliations:** Professor of Public Law and Legal Theory, University of Oxford & Head of Research, Bonavero Institute of Human Rights, Oxford; Professor of Law and Philosophy of Law, University of Oxford

**Keywords:** special jurisprudence, areas of law, philosophical foundations, legal theory

## Abstract

This article addresses three fundamental questions about a key phenomenon in special jurisprudence, ‘areas of law’: (i) what is an area of law; (ii) what are the consequences of dividing law into distinct areas; and (iii) what constitutes the foundations of an area of law. It claims that (i) ‘an area of law’ is a set of legal norms that are intersubjectively recognised by the legal complex as a subset of legal norms in a given jurisdiction; (ii) the sub-division of law into multiple areas matters to the content and scope of legal doctrine, to law’s perceived legitimacy and possibly to its effectiveness; and (iii) the search for the normative foundations of an area of law is typically an inquiry into its ‘aims’ or ‘functions’. This article systematically articulates, explains and answers these three questions *generally*, ie in relation to areas of law *as such*.

## 1. Introduction

Legal theorists, for the most part, used to confine themselves to the enterprise of understanding the nature of law and related phenomena *generally*. Only relatively recently has legal theory seen a surge in scholarly interest in theorising discrete ‘areas of law’ (variously described as ‘special jurisprudence’^1^ or ‘particular jurisprudence’,^2^ to contrast the field with general jurisprudence). General jurisprudence focuses on the nature of law and legal systems generally. It concerns itself with questions such as the conditions of a norm being a legal norm, the nature of legal obligation, whether the rule of law is inherently valuable, the nature of adjudication, and the possibility and implications of legal pluralism. The subject matter of theoretical inquiry in special jurisprudence, on the other hand, is normally a discrete *area* of law, such as labour law,^3^ discrimination law,^4^ tort law,^5^ family law,^6^ criminal law^7^ and constitutional law.^8^ While the interest in a theory of some areas of law—such as criminal law or contract law—has existed for a while, theoretical attention to most other areas of law is a relatively recent phenomenon.

The purpose of this article is to address three fundamental questions about the subject matter of a key phenomenon in special jurisprudence: ‘areas of law’. First, what is an area of law (the *ontological* question)? Second, what effects occur or are likely to occur as a result of the existence of areas of law (the *consequential* question)? Third, what, if anything, is the normative foundation of an area of law (the *foundational* question)?

The answers to these three questions matter both to the enterprise of special jurisprudence but also, to some extent, to legal practice. As to special jurisprudence, the first question concerns what is meant when a theorist offers a theory of *an area of law*—what makes it the case that a norm belongs to an area? This bears upon the issue of a theory’s fit with the law: to what extent, for example, is it acceptable to dismiss a particular norm as not really belonging to an area of law in order to argue that one’s theory fits the data? This depends on what makes it the case that a norm belongs to an area. As to legal practice, the law not infrequently makes the application of legal norms dependent upon area-based classifications. Legal education and legal thinking are pervaded by classification of norms into areas. Given the apparent importance of this phenomenon, obtaining a clearer understanding of its nature seems worthwhile. The second question has been considered in part by the literature on legal taxonomy, which has addressed the *values* served by classification of legal norms into categories.^9^ What our discussion adds is a focus on the likely empirical effects of the organisation of discrete legal norms into areas of law. The answer to the third question is primarily of theoretical concern. Despite the ubiquity of ‘functional’ analyses of areas of law or analyses that seek to identify the ‘aims’ of an area of law, the precise meaning of what it is to attribute ‘functions’ or ‘aims’ to an area remains obscure. We draw on the extensive philosophical literature on functions to illuminate this question. It follows from our analysis that a search for the normative foundations of an area of law is inherently a search for its functions or aims. So, our final section sheds light on the conceptual relationships between the functions, aims and foundations of areas of law.

Section 2 claims, in response to the ontological question, that ‘an area of law’ is a set of legal norms that are intersubjectively recognised by the legal complex in a given jurisdiction as a subset of legal norms in that jurisdiction.^10^ Thus, we offer a ‘social fact’ answer to the *existence question*, ie ‘in virtue of what does an area of law exist?’. In relation to the consequential question, section 3 shows that the sub-division of law into multiple areas matters to the content and scope of legal doctrine, to law’s perceived legitimacy and possibly to its effectiveness. Finally, in order to answer the foundational question, section 4 clarifies the sense in which areas of law have functions or aims, and shows that the search for the normative *foundations* of an area of law is typically an inquiry into its ‘aims’ or ‘functions’. All of these questions have been asked in a specific form with respect to particular areas of law before: on ontology, discrimination law and labour law theorists, for example, have worried about whether there is any unity to these areas of laws.^11^ With respect to the consequential question, doctrinal scholars make it their business to account for the interaction between areas of law and their discrete norms, whereas socio-legal scholars have examined the legitimacy and effectiveness of certain areas of law. And foundational accounts of discrete areas of law constitute the bulk of the literature in special jurisprudence. No one, however, has systematically articulated, explained and answered these three questions *generally*, ie in relation to areas of law as such.

## 2. The Ontological Question: What Is an ‘Area of Law’?

Characterised by Dworkin as ‘departments of law’,^12^ a feature of legal experience is the sub-division of the laws in a legal system into various ‘areas’. Here is our broad characterisation of the concept of an ‘area’ of law:

1. a subset of the legal norms in the system;
2. which is intersubjectively recognised by the legal complex in that system as a subset of such norms.

We take (1) to be relatively obvious. An area of law does not refer to the totality of the norms in the legal system, but a subset thereof. A reference to the totality of norms is simply to ‘the law’ in the system. (2) needs further comment. The reason for (2) can be brought out by asking whether there is recognised such a thing as, say, ‘Tuesday law’ (in, say, a jurisdiction like the UK). While there is likely to be a set of norms in all legal systems that applies to events on Tuesdays, this is clearly not sufficient for us to say that ‘Tuesday law’ exists as an area of law. Something more than mere legal regulation is needed.

Our example may be resisted on the basis that the norms that apply to Tuesdays are not geared *specifically* toward Tuesdays, and it is this, not the absence of social recognition, that makes us say there is no such thing as ‘Tuesday law’. But one can think of other, more plausible examples that *could* constitute an area of law, and perhaps even do so in some jurisdictions but not others. These might include insurance law (in, say, a country with laws governing insurance but an almost non-existent insurance market), cow protection law (which may yet emerge as an area of law in India, but is not currently recognised as such despite the prevalence of several cow protection statutes) or sexual harassment law (in a country that regulates it through norms scattered in its criminal and antidiscrimination codes, but does not yet have any special attention given to it by legal practice or education). Most jurisdictions tend to have laws regulating weights and measures, but we do not know of any that have an area of law for weights and measures. All of these could constitute an area of law; they just happen not to.

It is therefore insufficient for an object to be regulated by law for it to constitute a basis for identifying an area of law. Furthermore, the possibility that a subset of legal norms *could* be theorised as a logically coherent, normatively attractive and even practically useful unit is neither necessary nor sufficient for that subset to constitute an area of law. Suppose that a scholar proposes to theorise ‘Hohfeldian liabilities law’, meaning all of the legal norms that take the form of Hohfeldian liabilities correlated with Hohfeldian powers. This would involve theorisation of norms as diverse as powers to enforce debts and legislative powers, albeit with a logically coherent foundation (and, perhaps, a normative attractive one too). Irrespective, Hohfeldian liability law will not amount to an area of law unless it is recognised as such by the legal complex in a given jurisdiction.

There is nothing objectionable, of course, about theorising legal norms grouped under a classification that is not already employed in practice. Especially in the legal academy, there may be good pedagogic reasons for recognising what Cane called *expository classifications*: ‘the criteria of good expository classification are analytical elegance and economy, and facilitation of the teaching and learning of the law and of the process of finding out what the law is and of understanding what it means’.^13^ Hohfeldian liabilities law may well be a good expository category. Indeed, an important kind of legal scholarship seeks to *inaugurate* an area of law by developing a classification system of norms that is *not* already in existence. This is a plausible understanding of some taxonomic scholarship on the law of unjust enrichment. Prior to some point in the twentieth century, the law of unjust enrichment was not an area of law in England and Wales in the socially recognised sense we explain immediately below. Rather, there were many legal rules which (arguably) had a similar normative function or justification, but were not recognised in practice as bearing any relationship to each other. Scholars such as Peter Birks then argued that these diverse norms should be considered as forming a category, and should be brought together under an overarching framework.^14^ Today, it seems true to say that norms are classified together in practice as part of the law of unjust enrichment.

Thus, legal practitioners, educators and scholars play a performative role, in that they could attempt to create a new area of law, but their success will depend on the intersubjective recognition of this area as an area of law by a critical mass of actors in the relevant legal complex. Leon Green, in the early editions of his casebook on tort law, presented ‘materials for the study of judicial process as a whole rather than simply the integrity of some of its doctrines, theories, etc., [as] a departure from the usual case book study’.^15^ Instead of organising his material around doctrines such as ‘negligence’ or ‘nuisance’, as a typical textbook might, his *realist* chapters concerned ‘physicians, surgeons, hospitals’, ‘public service companies’, ‘traffic and transportation’ and so on. Green’s may be seen as an expository classification, but its sub-categories remain unrecognised by legal practice as sub-areas within tort law (at least from the practitioner’s internal point of view—this does not entail that Green’s exposition from the external/realist point of view is necessarily incorrect).

### A. Intersubjectively Recognised by the Legal Complex

An ‘area of law’ only exists, then, if it is intersubjectively recognised to exist by the ‘legal complex’, or some important subset of this, in a given society. The legal complex is a collective comprising legal actors involved in ‘drafting, representing, advising, judging, prosecuting, teaching, and writing law’.^16^ These actors can include judges, legislators, legal academics, private and government lawyers, civil servants, law reform movements, legal services regulators, law commissions, legal advisors, academic publishers, conference organisers, law journal editors, legal associations, court registries, casebook and textbook writers, case reporters, and the like. While legal scholars are part of the legal complex, they matter with respect to determining the existence of an area of law only to the extent that their research and teaching interacts with and influences the practice of law. Beyond this, which legal practitioners comprise the legal complex is mutable over time and space—in some jurisdictions or at certain times, bureaucrats may be key players, in others or at other times, they may not be.^17^ Similarly, which actors matter more and which matter less in determining the conceptual frameworks of the legal complex is also a contextual matter. Collectively, it is their ability to do certain things to and with law that matters. This feature marks a key distinction between general and special jurisprudence. As Les Green puts it, ‘Law is not itself a technical legal concept; it is part of ordinary social and political thought, and general jurisprudence begins with ordinary (lay) knowledge of law and society’.^18^ On the other hand, ‘special jurisprudence takes as its explananda *concepts within the law*, while general jurisprudence targets *the concept of law*’.^19^

Levmore, for example, persuasively argues that conceptually and normatively, ‘Deception may be as important a grouping as, say, torts’. Even so, because torts is a recognised area of law and deception is not,

when new cases arise in [torts], or in one of its subsets like trespass, judges try to fit them into the picture described by prior cases in the category … In contrast, judges have made no attempt to rationalize dispersed cases with deception as the common denominator.^20^

Areas of law exist, then, as what Raz calls ‘social forms’—ie forms of behaviour that are widely practised within that group.^21^ They are an *intersubjective* phenomenon, in the sense that their existence depends on their shared acceptance in the consciousness of at least a subset of persons comprising the legal complex in a given society. Not all details of their contents need to be shared—at most, some overlapping consensus about paradigm instances of the area in question will likely suffice—but as a ‘social form’,^22^ they cannot exist outside of some shared consciousness within this social group.

Their intersubjective character reveals a key feature of areas of law: while they are—*qua* areas of law—necessarily human artefacts, unlike particular legal norms they cannot simply be willed into existence by any individual or institution. To put the point differently, areas of law are not so much created by legal officials; they usually *evolve* into existence due to their recognition as areas of law by the legal complex. Individual actors can do things to hasten this evolution—start teaching a new course in law school, organise a conference, launch a specialist journal, specialise in its practice, publish a book on the topic, enact a comprehensive regulatory statute—all such attempts, even statutory ones, will fail to create an area of law unless a significant body of the legal complex as a whole accepts it as an area of law.

It is possible, for example, that despite the enactment of comprehensive human rights legislation, legal practice and the academy in a given jurisdiction continue to specialise in or teach its sub-fields as distinct areas of law, such as immigration and refugee law, anti-discrimination law, free speech law and so on. Or, there may be differences between jurisdictions: law schools in most common law jurisdictions tend to emphasise the commercial aspect of trusts in their courses on ‘commercial trusts’, whereas US law schools tend to focus on their family wealth management role, in courses typically called ‘Trusts and Estates’.^23^ When something has become an area of law is subject to the sorites paradox: just as we can never say for sure how many grains of sand are just about enough to qualify as a ‘heap’, it is impossible to put a number on the actors who must accept it as such before we can say that the legal complex has recognised it as an ‘area of law’. Food law may become an area of law in a particular jurisdiction, or it may not. Even if it does, we are unlikely to be able to pinpoint the precise moment in time when this transition happens.

### B. Recognition of Legal Norms as a Subset

There are many different possible bases to parcel out the law into areas. Here are some of the main possibilities.

*Regulatory object*. The specific object of legal attention is the most common basis for delineating areas of law. This could range from a type of person/entity (refugee law, company law, family law), a type of relationship (employment law, tenancy law), a social activity (banking law, construction law), an event (contract law), a category of things (antiques law, food law) and so on.

*Regulatory purpose*. Some areas of law are defined by the regulatory purpose they seek to achieve. Antidiscrimination law seeks to eradicate discrimination, consumer protection law seeks to protect consumers.

*Regulatory procedure*. Some areas of law are identified in a self-referential manner by reference to law’s own procedures, legal remedies or enforcement mechanisms. On one view, criminal law is the area of law that imposes norms whose breach is apt for punishment. Some areas of law, like evidence law, focus on aspects of the legal process.

Legal actors may disagree over what the classificatory basis is that underpins the area of law. For instance, some may believe that the law of equity is united not by its historical regulatory procedure (origins in the Courts of Chancery), but by its norms having a distinctive normative structure.^24^ What is essential to an area of law’s existence is that it constitutes a set of norms that are classified together for some reason by the legal complex—there is no need, so far as the existence of the area goes, that there also be any agreement on the correct basis for such recognition. This is shown by the fact that there is widespread convergence about what goes into textbooks on ‘equity’, even while the authors disagree about the classificatory basis on which the law of equity rests.

### C. Overlapping, Nested, Interactive, Dynamic

Different areas of law can interact and relate to each other in a variety of ways. Sometimes, a legal complex might classify law into a nested hierarchy of areas of law: at a broader level, law can be divided into ‘public law’ and ‘private law’ or ‘municipal law’ and ‘international law’, with numerous sub-areas populating each category. Constitutional law and the law of obligations may exist at an intermediate level of generality, accidents law and free speech law may well be recognised at an even lower level. Different categorisations can cut across each other: public law can be municipal or international, as can private law. Areas of law can also overlap: employment law and discrimination law have considerable overlaps in many jurisdictions. Even when they do not overlap, they can have closer affinities with some areas of law than others—perhaps because they belong to the same broader classification—and exert coherence pressures on ‘neighbouring’ areas of law. For example, commercial law is a lot more likely to be influenced by contract law than by administrative law. The same legal norms could belong to multiple areas of law, while others—such as legal norms regulating weights and measures—may belong to none. Finally, areas of law are temporally dynamic: they may come and go, their content may evolve over time, or they may merge into larger areas or be split into smaller ones.

### D. Objections to Our  View

It might first be objected that, on our view, the intersubjective recognition of a subset of norms in the legal system may be a brute social fact with no rational basis whatsoever. Our view is, indeed, consistent with this as a *conceptual* possibility. It seems to us correct to acknowledge the possibility that there may be, as a matter of social fact, areas of law which have no rational basis. That is, there may exist a classification of norms which has no value. If this is clearly the case, as may be so with our imaginary ‘Tuesday law’, then it is unlikely to be of theoretical interest as it would be clear that no rational explanation could be given for these norms as a group, even if particular norms within the group could be rationally explained. But if this possibility of an arbitrary, irrational, classification is ruled out *ex ante*, then those who theorise the (now recognised) ‘law of unjust enrichment’, and who—let us suppose, rightly—claim that it is a classification without value, are not theorising about an area of law at all.^25^ Rejecting the possibility of irrational areas of law makes it impossible to offer a critical normative account of a taxonomy recognised by a legal complex, since one denies the very existence of such areas in the first place.

A second objection is that inasmuch as we insist on the existence of areas of law being determined by social facts, our account assumes the truth of legal positivism, yet some prominent theorists of areas of law, such as Ernest Weinrib, do not subscribe to legal positivism. If our account is correct, the objection runs, then these theorists must have conflicting theoretical commitments: they are committed to a non-positivist account of law, yet, in identifying the subject of their theories, they must rely, if our view is correct, on a positivistic understanding of what makes an area of law exist. The objector might, for example, be tempted by the view that the concepts that pick out areas of law are, in Dworkin’s terms, ‘interpretive concepts’.^26^ This means that the content of the concept is determined by moral argument. For instance, the nature of democracy, according to Dworkin, depends upon what is the morally best interpretation of democratic practice.^27^

Our account does not, however, assume the truth of legal positivism. Legal positivism is a thesis about legal validity.^28^ It is not a thesis about the identification of subsets of legal norms that constitute legal areas. Someone could therefore consistently reject legal positivism yet endorse our view of the existence conditions of an area of law. Notably, Dworkin himself did not regard the concepts that marked out areas of law (or, in his terminology, ‘departments of law’) to be interpretive concepts.^29^ Although he insisted that ‘compartmentalization is a feature of legal practice no competent interpretation can ignore’, he also claimed that the boundaries of these ‘departments of law’ themselves are not interpretive phenomena but ‘based on tradition’.^30^ Sure, a judge may well seek to alter the traditional boundaries of classification for moral reasons. But these alterations will be effected, Dworkin accepts, only ‘If he persuades the profession of his view’.^31^ So, unlike his interpretive claim that what the law *is* cannot be determined without answering some normative questions, the existence of an area of law is, he concedes, determined by reference to a social fact concerning its recognition as such by the legal complex. This view seems coherent: Dworkin’s rejection of a positivist view of *legal validity* does not itself logically determine an answer to the question of what makes something an area of law.

Furthermore, the view that the existence of an area of law is an interpretive phenomenon seems implausible. Imagine that for some curious (perhaps religious) reason, a legal system sets up separate courts to deal with ‘Tuesday law’. All legally relevant events that take place on Tuesdays—crimes committed, contracts agreed upon, injuries inflicted—are litigated before these special theological courts. A separate set of the Bar, called Tuesday lawyers, develop specialism in Tuesday law. Law schools have special courses teaching Tuesday law. There is even a journal called *Tuesday Law Review* (published, oddly, on a Wednesday). For all that, no one can quite remember why Tuesday law developed in the first place, and there seems to be no contemporary reason for its continued existence in the now thoroughly secularised society. In fact, let us suppose there are very good, even overwhelming, reasons why Tuesday law should not exist as a separate area of law, but should be disaggregated and merged with the rest of criminal law, tort law, contract law and so on. Nonetheless, it is not at all clear to us in what sense one could claim that Tuesday law *would not still exist* as an area of law in that jurisdiction (until such time as its legal complex stops treating it as such). Whatever one’s views might be on the interpretive character of law, we suspect—with Dworkin—that an area of law is a social fact rather than an interpretive phenomenon.

In recognising the possibility of the existence of Tuesday law, we do not deny that it is as unlikely to be recognised as an area of law as other implausible categories that Levmore identified, such as laws concerning ‘cold-day facts’, ‘defendants named Smith’ and ‘red-colored property’.^32^ As social artefacts, legal complexes tend to recognise areas of law for instrumental reasons—to guide appropriately the development of law, to improve the expository accessibility of the law, to reap the epistemic benefits of specialisation: arbitrary categories like Tuesday law, even if they did come about for some quirky reason, are likely to die away when they stop serving any helpful purpose.^33^ Thus, Easterbrook argued against creating an area of ‘horse law’ for pedagogic purposes:

Lots of cases deal with sales of horses; others deal with people kicked by horses; still more deal with the licensing and racing of horses, or with the care veterinarians give to horses, or with prizes at horse shows. Any effort to collect these strands into a course on ‘The Law of the Horse’ is doomed to be shallow and to miss unifying principles.^34^

But the irrationality of Tuesday law (or horse law, for that matter^35^)—if it *is* recognised as an area of law in some jurisdiction—is a reason to discard it; it cannot, in itself, refute the existence of that area of law until the legal complex stops recognising it as such.

We admit, however, that while the existence of an area of law is a social fact, its precise scope and boundary may require normative determination, especially when the area of law is recognised by legal authorities so that a legal issue turns upon such a determination. Social facts determine whether an area of law exists and what paradigmatic norms it contains; they cannot settle all disputes about the allocation of complex norms or causes of actions to particular areas of law. When such determination becomes relevant for practical or theoretical purposes, some sort of normative judgment may be required. Appeals to the purported foundations (ie aims/functions) of the candidate areas of law are inevitable in such cases.

## 3. The Consequential Question: Do Areas of Law Matter?

Areas of law are a ubiquitous feature of legal systems. But does this departmentalisation of law matter? Were it feasible to have one, would an undepartmentalised legal system follow a different developmental trajectory than one with the same substantive norms, divided into multiple areas of law? Does it matter that a legal system divides its norms this way rather than that? What difference does it make if we assign a legal norm to one area of law rather than another? In this section, we will show that the sub-division of law into multiple areas matters to the content and scope of legal doctrine, law’s perceived legitimacy and possibly its effectiveness.

First, classification of law into multiple areas matters to legal outcomes, because a central reason for developing legally recognised areas of law is that the classification can be relied upon to generate legal conclusions.^36^ Judges rely upon the classification of a norm as a norm of tort law *as a doctrinal matter* to generate further legal conclusions, for instance, as to the availability of certain remedies, applicable proof rules or applicable defences. In Peter Cane’s terminology, these are *dispositive* categorisations: they are employed in legal reasoning to generate legal conclusions.^37^ In short, insofar as a classificatory scheme is treated as a *dispositive* rather than a merely *expository* scheme, it may have a significant impact upon the development of the law. Here is one example from the conflict of laws.^38^ In *Anton v Bartolo* (the Maltese marriage case) decided by the Court of Appeal at Algiers in 1889, a question concerning succession law was to be governed by the law where the land in question was located (in this case, France), whereas one about matrimonial rights was to be adjudicated under the law of the jurisdiction where the couple were domiciled (here, Malta). However, the facts themselves were treated by French law as giving rise to a cause of action under the law of succession, whereas under Maltese law they would have given rise to one under matrimonial law.^39^ Another example may be the different ways in which contract law and the law on professional ethics in some jurisdictions may treat defective representation that a lawyer provides to her client. Which area of law the cause of action is allocated to therefore determines the applicable legal regime in such cases.

Secondly, it is not only areas of law that qualify as dispositive classificiations that affect legal doctrine: the mere recognition (and, therefore, the constitution) of an area of law by the legal complex has important consequences for the content of its norms even if the area of law does not qualify as a dispositive classification recognised by legal doctrine. For example, even if consumer law—unlike, say, tort law—is not dispositive in the sense that the recognition of a norm or a cause of action as falling within consumer law dictates certain legal consequences (eg a certain type of remedy), the mere existence of consumer law as an area of law in a jurisdiction is likely to have consequences for its norms.^40^ Once an area of law is recognised by the legal complex (even if not by legal doctrine), various actors start attempting to lend it some degree of coherence. This is especially the case when the legal area becomes recognised by legal authorities. Much training in law schools and litigation in appellate courtrooms (at least in common law systems) concern smoothing out law’s eccentricities and inconsistencies, for part of the justification of law rests on its being coherent. This aspiration for coherence is even more pronounced within an area of law because when actors become specialists in an area, lawyers notice and point out incoherence more readily (at least when it is in their client’s interests) and judges start tending to iron it out.^41^ These coherence-making efforts may not always succeed: it may well be that a theoretical analysis ends up with a sceptical conclusion—that there is nothing that unites an area of law (except, of course, its social recognition as an area of law).^42^

However, given the practical and academic aspiration to coherence in law, even a disparate collection of legal norms is likely, over time, to come to acquire some measure of doctrinal unity. In adjudication, norms within an area of law tend to exert stronger interpretive pressures on consanguineous norms than those outside it because of their presumed or desired normative coherence, at least when the area of law is taken to be founded upon a particular aim or function.^43^ Legislatively, law codes tend to cover (sometimes, eventually, create) *an* area of law, whereas individual statutes interact with (ie amend, repeal, modify the operation of, etc) other statutes within the same area of law far more frequently than those outside that area. Specific areas of law are sometimes given bespoke legal institutions for their enforcement, including specialist courts, tribunals, commissions, bureaucracies and so on. This is an empirical hypothesis about the nature of law and legal practice in a reasonably well-functioning common-law based legal system—once an area of law is recognised, a pull towards coherence within that area of law will almost certainly follow.^44^ This is compatible with the possibility that increased coherence *within* an area may well result in greater divergence *between* doctrines in different areas of law, and therefore between doctrines in the legal system more generally.

Thirdly, legal actors, especially academics and appellate court judges, speak not only of doctrinal coherence, but also of *foundational* coherence (indeed, doctrinal coherence is typically brought about by appealing to a coherent normative foundation).^45^ The recognition of an area of law by the legal complex typically entails the assumption that it exists to serve some identifiable normative aim or function (or set of aims or functions). The assumption may be mistaken when the area of law comes about, but it is self-fulfilling: the fact that the legal complex assumes some foundational unity frequently leads to the area acquiring one. As MacCormick puts it,

the coherence of norms is a matter of their making sense by being rationally related to a set, instrumentally or intrinsically, either to the realisation of some common value or values; or to the fulfilment of some common principle or principles.^46^

It is this *foundational* coherence that lends an area of law a sense of identity or unity, and makes it intelligible *qua* an area of law (rather than a random collection of legal norms).

Fourthly, once there is broad consensus over an area of law’s foundation—in the sense that a critical mass of relevant actors in the legal complex accept that it is founded upon a particular aim or function—it allows legal systems to embed the value underpinning this foundational aim or function within the ideological commitments of their polities, or at least of the legal system itself. A jurisdiction which lacks the comprehensive organisational categories of ‘private law’ and ‘public law’ entirely, and instead has as its fundamental building blocks ‘law of individuals’, ‘law of corporations’ and ‘law of the state’ may not start with the neoliberal assumption that the law of corporations is simply a special case of ‘private law’.^47^ A legal system that recognises an area of ‘discrimination law’ will likely regulate discrimination differently from one which does not, even if the latter has antidiscrimination norms scattered around its statutes in different areas of law (constitutional law, employment law and so on).

These ideological underpinnings matter because areas of law are hard to establish; they are even harder to abolish, especially after they have existed for a while. Coherence-seeking, both doctrinal and normative, is what areas of law do because the aspiration of every area of law is to become stable over time, with doctrinal contours that are more or less settled, and about whose normative foundations there exists a broad consensus. A long-standing regime of property law is a paradigmatic example of a mature and stable area of law: hard cases, when they arise in such an area, will normally be resolved by analogical reasoning with existing doctrine or occasionally by first principles that are taken as settled. Once a system establishes a stable area of law based on a particular ideological foundation, the ideology itself can recede into the background while still operating powerfully, while coherence-led analyses can suffice to resolve hard cases. If this regime of property law, say, was underpinned by a strong libertarian aim, it is likely to make it very difficult for that jurisdiction to undertake meaningful land redistribution. On the other hand, relatively new areas of law whose aims or functions are more controversial (and, therefore, more visible)—take discrimination law, for example—are more readily repurposed for different agendas.^48^ In these less mature areas, judges will frequently need to make (contested) foundational claims to resolve hard cases.

A consensus over its foundations lends an area of law an ideological stability, which may be a key facilitator of the law’s normative force and, therefore, its perceived legitimacy. On the other hand, this very ideological underpinning (because it can operate silently and somewhat invisibly) can mask normative choices as if they were technical and value-neutral. For example, as Harcourt argues, the deployment of cost–benefit analyses within regulatory systems—themselves usually demarcated by areas of law—requires the determination of the scope of the system, its foundational values and the choice of alternative enforcement measures which will maximise these values.^49^ All of these moves are normative and political, but can be presented as technical rationality. For example, Harcourt argues that applying cost–benefit analysis to the ‘criminal justice system’ rather than the ‘racial equality system’—presumably because criminal justice is an area of law in that jurisdiction but race equality law is not, or not one with the same salience—would prioritise crime prevention over antidiscrimination.^50^ Even worse, it will do so because it takes the existence of criminal justice law rather than race equality law as an area of law to be a logical rather than a constructed feature of the legal system, and one that is normatively (more) consequential. To return to a previous example, it may be that the public–private divide is so successful in manifesting itself in vast swathes of legal norms because it presents itself as a technical, rather than an ideological, distinction.

A further consequence of our claim that areas of law seek foundational coherence is that different areas of law within the same legal system can be founded upon distinct, even mutually incoherent, normative aims or functions. It is possible, for example, that the area of discrimination law within a given legal system is justifiable by a redistributive egalitarian conception of justice, whereas the area of property law in the same jurisdiction is explainable through libertarian ideals of just allocation of resources. Thus, areas of law—even when internally coherent—may well play a key role in the ideological heterogeneity of legal systems, taken as a whole.

Fifthly, and most speculatively, it may well be the case that the actual operation of legal norms in the world is dependent on the type of area of law they belong to. It is at least plausible that discrete areas of law in a given jurisdiction display distinct characteristics in terms of their degree of convergence with global legal standards, their level of compliance and enforcement, their perceived legitimacy among legal subjects, the dynamism or stability of their norms, their political salience, the distinct preservative or reformatory pressures that they experience and so on. Are there patterns of behaviour? Do some types of areas of laws (say those organised by a particular legal transaction, say contract law) tend to have different characteristics from others (perhaps those based on certain persons, say family law)? Does it matter to its real-world impact whether an area of law has a broad or a narrow scope, a dedicated or a generalist enforcement agency, has existed for a long time or is relatively novel, or concerns the economy, society or politics? Are specialist judges more likely to be overzealous in enforcing the specific aims of their particular area of law? Perhaps areas of law of narrow scope (‘aviation law’) are more amenable to industry capture than those of broad scope (‘transport law’)? Areas of law—as a general legal phenomenon—may lend themselves not only to analytical and normative theorising, but also empirical research.

For all these reasons, whether and how legal norms are divided into distinct areas matters to the content, the perceived legitimacy and likely the operation of the law. Having looked at the ontological and consequential questions, we can finally turn our attention to the foundational question in relation to areas of law.

## 4. The Foundational Question: How Do Areas of Law Have Functions or Aims?

In this section, we will explain what it means to offer an account of the ‘functions’ and ‘aims’ of an area of law. It follows from our analysis, as we will show, that an account of the normative ‘foundations’ of an area of law will almost certainly be a normative theory that identifies its ‘functions’ or its ‘aims’.

### A. Functions

The concept of a ‘function’ is the subject of a rich philosophical literature, which, despite the prominence of ‘functional’ accounts of areas of law, is rarely drawn upon in special jurisprudence.^51^ It is impossible for us fully to defend a theory of functions here, but we can articulate some important distinctions. Here are two senses in which F might be described as a function of an object X:

- X is used for F; or
- X does F and exists or is maintained because of its contribution to F.

Consider (i). Suppose that a person uses a table as a weightlifting device. One view is that this has no impact on the ‘function’ of the table. The table’s function is still to hold objects above the ground, but it is being used for some other purpose. While the table *functions* as a weightlifting device, its *function* is to hold objects above ground. It is, however, intelligible to say that the *table functions in this context* as a weight.^52^ Hence (i). Notice that the word ‘function’ is more appropriately used as a verb in these, more peripheral, usages; ‘function’ as a noun tends to describe its more paradigmatic uses,^53^ which we will consider next.

If the function of an object depended entirely upon what it is used to achieve, implicating the idea of a *user*, it would be puzzling how non-artefacts, like hearts, could have functions. But it is intelligible to say that the ‘function’ of the heart is to pump blood. It is initially tempting to say that the function of natural objects is simply *what those things typically do*. The difficulty is that the heart typically does many things which are not its functions: it makes a throbbing noise, for instance. There is a difference, then, between what something *does* and its *function(s)*. (ii) aims to capture this. Pumping blood is something the heart does and something which explains why it exists: by virtue of the causal process of natural selection, we would not have hearts if they did not pump blood. But we would still have hearts even if they were not noisy. (ii) also captures the sense in which artefacts have functions. A corkscrew’s function is to remove corks. That is something corkscrews do *and* why we have corkscrews. (ii) captures the paradigmatic use of the word ‘function’ as a noun: the *function* of corkscrews is to remove corks, even though a corkscrew can *function* as a paperweight (notice the verb form associated with (i)). (ii) allows for the possibility of an object having multiple functions: there may be multiple reasons why we have that object. The function of windows is to let in light as well as to allow the circulation of outside air into a building. (ii) also allows that the function of an object can change: the reason why we *maintain* an object in existence could be different from the reason why the object was *brought into existence* (for example, certain drugs were first created to treat disease A, but their current function is to treat disease B).

This account of a ‘function’ illuminates talk of functions in law, too. Consider the non-paradigmatic case (i). It is clearly intelligible to ask what an area of law is *used for*. If the law against defamation is used to silence critics of the ruling party, it may be accurate to say that defamation law *functions* or *is functioning* as a tool to silence such critics. This allows for the possibility that defamation law is being used in the same way as a table is used when it is used as a weight. It is this verbal (rather than nominal) sense of function which is often at issue in empirical accounts of areas of law.

Consider now (ii). (ii) makes sense of the idea that much as a heart can have a function without it being designed to do something or anyone intentionally using it for that thing, so too can an area of law. (ii) helps to illuminate how this could be so. Consider deterrence in tort law. Suppose that a new legal system establishes what would normally be accepted to be tort law *solely in order to create optimal deterrence*. In a sense, then, deterrence can be described as a function of this system of tort law. Even so, a corrective justice theorist may still be right that deterrence is not a function of tort law in a sense picked out by (ii). Even if the sole *causal* reason for establishing the area was officials’ belief that tort law deters, it does not follow that this is a function of tort law in the sense of function picked out by (ii). Rather, it could still be the case that deterrence is not a *normative reason* for having tort law, so that tort law deters does not contribute to the rational case for its existence or maintenance.^54^ If considerations of corrective justice provide normative reasons for having an area of law with those features, then we can say that tort law has this function—*even if* these normative reasons played no causal role in the establishment of the area of law.

Some may resist this last point because it allows that an area of law could have a function which arises *entirely* as a matter of coincidence. The stars may align so that judges produce a recognisable system of tort law, entirely unmotivated by considerations of corrective justice, yet, on this understanding of function, corrective justice is a function of tort law. An analogy: suppose that the random rock formation in a river causes the downstream river delta to be much broader than it would otherwise be. Suppose also that the downstream water cycles back around in such a way as to cause the rock formation to be in its current location. We might be reluctant to say that the function of the rock formation is to broaden the river delta, even though this is something it does, and something which explains the continued presence of the rock formation. This is perhaps why McBride argues that a theoretical account of private law must identify some *mechanism* that explains how the law could have been developed to reflect the normative reasons that explain it.^55^ This suggests that a further distinction may be useful: there is a distinction between normative reasons that contribute towards the rational case for the existence of an area of law and normative reasons that, perhaps only implicitly, motivate or motivated officials to create and develop the area. We could draw this distinction by distinguishing between *normative-explanatory* functions (the former) and *institutionally embedded* functions (the latter).

A complication surrounding the notion of function in (ii) should be addressed. It seems possible for an object, and an area of law, to have a function yet not to perform that function, or to be very inept at performing the function. If a vacuum cleaner’s wire is severed, it is presently unable to suck up dust, yet the vacuum cleaner’s function is still to suck up dust. Perhaps constitutional law, as it operates in the world, does not in fact constrain state power effectively, but it could still conceptually have as one of its functions the constraining of state power.^56^ In other words, it *malfunctions*.

The difficulty is that (ii) refers to X as actually performing the function—‘X does F’—which seems to rule out attributing function F, when X does not in fact F. There are at least two possible responses to this difficulty. First, so long as X does F in some measure, then it will be possible to attribute the function to the object. If constitutional law does, to some extent, constrain state power, and this is a reason for having it, then the function attribution stands. Second, if X *would* F in ordinary circumstances, or in circumstances which are close to current circumstances, and this modal property of X is a reason for having X, then this may be sufficient to attribute F as a function of X.^57^ If there is a nearby possible world in which the vacuum cleaner’s wire is fixed and the machine’s suction is restored, then this can be attributed as a function. Suppose that a particular system’s constitutional law could constrain the state by modifying some relatively minor aspects; then constraining state power can still be said to be the function of constitutional law. (ii) allows for the possibility that Xs credibly aspire to do F. Credibility, of course, is a matter of degree—after a point, it is legitimate to characterise constitutions that are wholly inept in their constraining function as ‘façade constitutions’.^58^ Furthermore, (ii) even permits a detached normative theorist to argue that F is the function of an area of law without committing herself to whether F is desirable.^59^ It is entirely possible to hold the view that one of the functions of liberal rights is to express the sanctity of private property without also believing that such expression of private property’s sanctity is at all desirable.^60^

In the theory of private law, there is sometimes thought to be something inherently problematic about an explanation of an area of law in terms of its *functions*. Weinrib famously argues against accounts of tort that understand its function to be the pursuit of economic efficiency.^61^ It is not always clear, however, whether Weinrib objects to functional explanation *as such* or simply to an analysis of tort law in terms of *particular* ‘external’ functions. We doubt that there can be an objection to a functional explanation of areas of law *as such*. Because it is a human artefact, it always makes sense to ask of an area of law: *why do we have it?*^62^ The answer to this question, so far as it is not seeking merely a causal explanation, is an account of a function of the area. It would still make sense (for Weinrib) to say then that the function of tort law—in the thin sense that we are using the term ‘function’—is to reflect and effectuate corrective justice. Nor does our thin sense of the term imply that if F is a function of an area of law, F must be *maximised* in some way. In our sense, then, even Weinrib’s self-avowedly ‘anti-functionalist’ theory that tort law constitutes and dispenses corrective justice can reasonably be understood as a claim about the *function* of tort law.^63^

We can see now that an account of an area of law’s functions, so far as it tracks (ii), is simply a kind of detached or committed normative theory of that area. Since functions, in this sense, are reasons for having or maintaining particular areas of law (or particular rules therein), theories of functions are necessarily theories of the normative reasons for having the area of law.

### B. *Aims*

Having explained functions, we can turn now to aims. An agent, X, has an *aim* if and only if:^64^

X has, or can reasonably be imputed to have, an intention or intention-like mental state to achieve A.

X might be wholly deluded in thinking that X has any reasonable prospect of actually achieving A but, nonetheless, if X intends A, then A is X’s aim.

How, then, can an area of law be said to have an *aim*? There are three possibilities. First, areas of law could themselves be said to be collective agents with shared collective intentions to achieve ends. Understood simply as a socially recognised subset of norms, this seems self-evidently implausible. Second, the aims of an area of law may simply be an elliptical statement of the aims of some (not necessarily identified) agent, or set of agents, not itself identical with the area of law. On this view, statements like ‘discrimination law’s aim is to reduce the relative disadvantage of protected groups’ are akin to statements like ‘the bomb’s aim was to kill’. The latter sentence is best understood as elliptically referring to the aims of the agent who deployed the bomb, and not the bomb itself, which is the means adopted by the agent to achieve her aim. The sentence will be more accurate in the passive voice: ‘the bomb *was aimed to* kill’. Strictly speaking, on this view, an area of law itself cannot have any aims—when we speak of the aims *of* an area of law, we speak elliptically of the aims of whichever agent(s) we understand to be employing it as its means. This is reflected in (i) below. A third, related possibility is that an area of law has an aim if it would be rational to *impute* that aim to those agents relevantly connected to the area, even if the aim is not consciously adopted by those agents. If some practice can only be understood as instrumentally reason-governed if it is in the service of some aim A, then one may impute that aim to the practice. The explanation can be detached or committed, depending on the theorist’s own normative attitude towards the imputed aim. This is captured in (ii) below. An account of the aim A of an area of law could, then, refer to a number of things:

- an empirical account of the actual intention or intention-like attitudes of those employing S as a means to achieve A; or
- a detached or committed normative theory that the imputation of A to the agents employing S as a means could explain why S exists or is maintained, whether or not A has in fact been adopted by such agents.

Consider now the relationship between *functions* and *aims*. Function in the first sense, ‘what something is used for’, corresponds to the aim of some agent in using that thing. Function in the second sense, as what an area does or would do that explains its existence, need not correspond to any agent’s intention. The area’s function, in this sense, is one of its inherent properties, which depends on what rationally explains the area. However, this sense of function corresponds to the third way in which an area might be said to have an aim: when it is rational to impute that aim to the agents relevantly connected to the area. If there is only one way of explaining the area rationally, then one can rationally impute this aim to the area, even if no agent adopts that aim. In the context of some areas of law, then, where the identity of any agent is often obscure, the distinction between functions and aims may appear to be much of a muchness.

A further distinction is that aims can be realised to a greater or lesser extent, and quickly or slowly. It is not unreasonable to adopt aims that one knows can never be fully realised. Aspiring towards it and getting closer to it may yet be worthwhile.^65^ An agent can have an aim (as in (i) above) even if her chosen means of pursuing it is wholly unsuitable and is never likely to succeed. On the other hand, an object can be more or less suitable for performing a function, but—as our discussion of malfunction above demonstrated—below a certain threshold of ineptness we are better off characterising it as either dysfunctional (if it ever was functional) or unrelated to the function at hand.

The relevance of the distinction between aims and functions can also be appreciated in terms of normative analysis. If an area of law does not—or, with minor modifications, cannot—perform a certain task, the performance of that task simply cannot be its function (in the paradigm sense of ‘function’: (ii) above). If a critical claim that what constitutional law in fact does is cloak illegitimate power with a veneer of legitimacy while failing entirely to meaningfully constrain it is correct, constraining power simply cannot be a *function* of constitutional law. It can still be the *aim* that the relevant agent has/should have, albeit one that is being pursued by inappropriate means. A measure of real-world success in suitable circumstances is a precondition for attributing a function; it is not so for an agent having a certain aim (as in (i) above).

Finally, while we accept that the growing literature on ‘philosophical foundations’ of areas of law tends to apply the term loosely to almost any enterprise in special jurisprudence, one important conception of the normative *foundations* of an area of law is an account of the normative reasons which justify having that area. It may, then, consist of an account of its *functions* (ii) or *aims* (ii). More clearly, any of the following is a foundational account of an area (S) of law in terms of a function F or aim A:

- S does F or would F if minor changes were made to S or its circumstances of operation, and S exists or is maintained because it does or would F;
- the imputation of A to the agents employing S as a means could explain why S exists or is maintained, whether or not A has in fact been adopted by such agents.

Many disputes in special jurisprudence concern the identification of the right function of an area of law or the actual or reasonably imputable aim of its authors/administrators. We end this discussion by noting that an inquiry into the normative foundations of an area of law is only one important sense in which a foundational theory can be pursued. It is possible for instance to examine the conceptual foundations of an area of law: an analysis of the law of property, for example, might identify the necessary and sufficient conditions for a right to qualify as a property right, or the characteristic features of property rights.

## 5. Conclusion

This article is aimed at advancing the as yet relatively limited *general* discussion about special jurisprudence. Ontologically, we have shown that whether an area of law exists in a jurisdiction is entirely a matter of its intersubjective recognition as such by the legal complex, although the determination of its boundaries may entail normative considerations. Consequently, once an area of law comes into existence, its existence has real implications on legal doctrine, law’s perceived legitimacy and its effectiveness. Finally, we have given an account of what it is to elucidate the normative foundations of an area of law by explaining what it is for an area of law to have a function or aim. These are some of the, but of course not the only, salient general theoretical issues relevant to the field of special jurisprudence.

## Footnotes

1 L Green, ‘General Jurisprudence: A 25th Anniversary Essay’ (2005) 25 ojls 565, 565; J Waldron, ‘Can There Be a Democratic Jurisprudence?’ (2009) 58 Emory LJ 675, 678.

2 For a history of the terms ‘general’ and ‘particular’ jurisprudence, see W Twining, *Law in Context: Enlarging a Discipline* (Clarendon Press 1997) ch 8. The terms, and a broadly connected distinction, are traceable to Jeremy Bentham, but their meaning is different.

3 A Bogg, ‘Labour, Love, and Futility: Philosophical Perspectives on Labour Law’ (2017) 33 International Journal of Comparative Labour Law and Industrial Relations 7; H Collins and others, ‘Does Labour Law Need Philosophical Foundations?’ in H Collins, G Lester and V Mantouvalou (eds), *Philosophical Foundations of Labour Law* (OUP 2018).

4 D Hellman and S Moreau (eds), *Philosophical Foundations of Discrimination Law* (OUP 2013); H Collins and T Khaitan (eds), *Foundations of Indirect Discrimination Law* (Hart Publishing 2018).

5 D Owen, *Philosophical Foundations of Tort Law* (Clarendon Press 1995); J Oberdiek (ed), *Philosophical Foundations of the Law of Torts* (OUP 2014).

6 E Brake and L Ferguson (eds), *Philosophical Foundations of Children’s and Family Law* (OUP 2018).

7 J Gardner, *Offences and Defences: Selected Essays in the Philosophy of Criminal Law* (OUP 2007); V Tadros, *Wrongs and Crimes* (OUP 2016).

8 DA Strauss, ‘What Is Constitutional Theory?’ (1999) 87 CLR 581; J Varuhas, ‘Taxonomy and Public Law’ in M Elliott, J Varuhas and S Wilson Stark (eds), The Unity of Public Law? (Hart Publishing 2018).

9 The literature is enormous. See eg P Birks, ‘Definition and Division: A Meditation on Institutes 3.13’ in P Birks (ed), *The Classification of Obligations* (OUP 1997); H Dagan, ‘Legal Realism and the Taxonomy of Private Law’ in C Rickett and R Grantham (eds), *Structure and Justification in Private Law* (Hart Publishing 2008) 147; S Smith, ‘Taking Law Seriously’ (2000) 50 UTLJ 241; E Sherwin, ‘Legal Positivism and the Taxonomy of Private Law’ in Rickett and Grantham (ibid) 115–21; S Waddams, *Dimensions of Private Law: Categories and Concepts in Anglo-American Legal Reasoning* (CUP 2003); C Webb, ‘Treating Like Cases Alike: Principle and Classification in Private Law’ in A Robertson and Hang Wu Tang (eds), *The Goals of Private Law* (Hart Publishing 2009) 216–17.

10 On the concept of the ‘legal complex’, see the discussion in s 4 below.

11 T Khaitan, ‘Prelude to a Theory of Discrimination Law’ in Hellman and Moreau (n 4) 138; A Bogg and others (eds), *The Autonomy of Labour Law* (Bloomsbury 2015).

12 R Dworkin, *Law’s Empire* (Fontana 1986).

13 P Cane, *The Anatomy of Tort Law* (Hart Publishing 1997) 198–202.

14 See eg P Birks, *Introduction to the Law of Unjust Enrichment* (Clarendon Press 1988).

15 L Green, *The Judicial Process in Tort Cases* (2nd edn, West Publishing House 1939) ix. See also, J Jolowicz, ‘Fact Based Classfication of Law’ in J Jolowicz (ed), *The Division and Classification of the Law* (Butterworths 1970) for a comprehensive proposal of a ‘fact-based’ classification of an entire legal system.

16 L Karpik and TC Halliday, ‘The Legal Complex’ (2011) 7 Annual Review of Law and Social Science 217, 220-221.

17 ibid 221.

18 L Green, ‘Gender and the Analytical Jurisprudential Mind’ (2020) 83 MLR 893, 894.

19 ibid 895 (emphases in the original).

20 S Levmore, ‘A Theory of Deception and Then of Common Law Categories’ (2007) 85 Tex L Rev 1359, 1361.

21 See J Raz, *The Morality of Freedom* (Clarendon Press 1986).

22 ibid 308–10. Raz understands social forms to be ‘forms of behaviour which are … widely practised in society’ or ‘common social forms of action’. Social forms have ‘internal richness and complexity’ and the degree to which a practice has to be shared, and by whom, are open questions.

23 T Gallanis, ‘Commercial Trusts in US Legal Thought: Historical Puzzles and Future Directions’ (2020) 88 U Cin L Rev 842, 847.

24 B McFarlane and R Stevens, ‘What’s Special About Equity? Rights about Rights’ in D Klimchuk and others (eds), *Philosophical Foundations of Equity* (OUP 2020). More generally, disagreements about classifications in law persist because of the different purposes that different actors have for making a classification, the multi-dimensionality of the bases of law’s classification, and–as we will see in the next section, the overlapping, nested and inexhaustive nature of legal classifications: W Twining, K O’Donovan & A Paliwala, ‘Ernie and the Centipede’ in J Jolowicz (n 15). See also, D Lasok, ‘Reflections on the Problem of the Classification of Law’ ibid 70-71.

25 For a denial of the value of the category ‘unjust enrichment’, see R Stevens, ‘The Unjust Enrichment Disaster’ (2018) 134 LQR 574.

26 See Dworkin, *Law’s Empire* (n 12) ch 3; R Dworkin, *Justice for Hedgehogs* (Harvard UP 2011) chs 7 and 8.

27 R Dworkin, ‘Hart’s Postscript and the Character of Political Philosophy’ (2004) 24 ojls 1.

28 As carefully explained by J Gardner, ‘Legal Positivism 5 ½ Myths’ (2001) 46 Am J Juris 199.

29 See Dworkin, *Law’s Empire* (n 12) 250–4.

30 ibid 251–2.

31 ibid 254.

32 Levmore (n 20) 1377.

33 To be sure, if Tuesdays did still have a religious significance in that population, then Tuesday Law would still serve the practical purpose of religious observance.

34 F Easterbrook, ‘Cyberspace and the Law of the Horse’ (1996) U Chi Legal F 207.

35 ‘Horse law’ might have more going for it than Easterbrook allows. One could say similar things about ‘consumer law’, but it may be helpful to consider the norms concerning consumers together for certain purposes, notably, to understand if these norms work overall to the benefit of consumers or whether they undermine each other.

36 See especially Webb (n 9) 216–17.

37 Cane (n 13) 198–202.

38 See generally P Torremans and others (eds), *Cheshire, North, and Fawcett: Private International Law* (15th edn, OUP 2017) 41–2.

39 ibid 43.

40 Note that in this example, consumer law—while not dispositive—is not merely *expository* either, since it exists as an area of law, by definition, if it is intersubjectively recognised by a critical mass of actors in the legal complex, and not just by a few scholars for pedagogic purposes.

41 Indeed, at least part of the value of having ‘areas’ of law is to identify incoherence more easily than would otherwise be the case.

42 George Rutherglen thinks that discrimination law has no unity nor identity. See GA Rutherglen, ‘Concrete or Abstract Conceptions of Discrimination’ in Hellman and Moreau (n 4) 115.

43 Dworkin, *Law’s Empire* (n 12) 402–3.

44 Our claim is distinct from what Raz calls the ‘constitutive coherence theories of law and adjudication’, which is his characterisation of Dworkin’s interpretivism. See J Raz, *Ethics in the Public Domain* (Clarendon Press 1995) ch 13.

45 See the next section for details.

46 N MacCormick, ‘Coherence in Legal Justification’ in A Peczenik and others (eds), *Theory of Legal Science: Proceedings of the Conference on Legal Theory and Philosophy of Science Lund, Sweden, December 11–14, 1983*, SYLI vol 176 (Springer 1984) (emphases in the original ignored).

47 See Julian Sempill, ‘What Rendered Ancient Tyrants Detestable: The Rule of Law and the Constitution of Corporate Power’ (2018) 10 HJRL 219. Also, Halley and Rittich show how the distinction between contract law and family law deeply entrenched the ideology-inflected differential legal treatment of relations within the market and the family: J Halley and K Rittich, ‘Critical Directions in Comparative Family Law: Genealogies and Contemporary Studies of Family Law Exceptionalism’ (2010) 58 Am J Comp L 753.

48 Jack Balkin and Reva Seigel, ‘The American Civil Rights Tradition: Anticlassification or Antisubordination?’ (2003) 58(9) U Miami L Rev 9.

49 B Harcourt, ‘The Systems Fallacy, A Genealogy and Critique of Public Policy and Cost–Benefit Analysis’ (2018) 47 JLS 419.

50 ibid 421. For a real-world illustration of the issue, see P Karlan, ‘Race, Rights, and Remedies in Criminal Adjudication’ (1998) 96 Mich L Rev 2001.

51 See eg L Wright, ‘Functions’ (1973) 82 The Philosophical Review 139; R Cummins, ‘Functional Analysis’ (1975) 72 Journal of Philosophy 741; P Godfrey-Smith, ‘A Modern History Theory of Functions’ (1994) 28 Nous 344; B Nanay, ‘A Modal Theory of Function’ (2010) 107 Journal of Philosophy 412. There are well-known discussions of functional explanation in general jurisprudence, see eg M Moore, ‘Law as a Functional Kind’ in RP George (ed), *Natural Law Theory: Contemporary Essays* (Clarendon Press 1992). For a useful discussion, see K Ehrenberg, *The Functions of Law* (OUP 2016), ch 2.

52 For this contrast, see R Millikan, *Language, Thought, and Other Biological Categories: New Foundations for Realism* (MIT Press 1984) 17, introducing the notion of a *proper* function, to distinguish the particular use to which an object is put from its intended or designed-for use.

53 See Wright (n 51) 140.

54 For doubts about the view that deterrence is *never* a normative reason for private law rules, see S Steel, ‘Deterrence in Private Law’ in H Psarras and S Steel (eds), *Private Law and Practical Reason* (OUP forthcoming).

55 NJ McBride, *The Humanity of Private Law: Part 1. Explanation* (Hart Publishing 2019).

56 G Sartori, ‘Constitutionalism: A Preliminary Discussion’ (1962) 56 American Political Science Review 853, 855.

57 See Nanay (n 51).

58 Sartori (n 56) 861.

59 On ‘detached’ and ‘committed’ theorising, see T Khaitan and S Steel, ‘Theorising Areas of Law’ (unpublished manuscript).

60 A third possibility is to distinguish between types and tokens. A particular token X may have F as its function, even if X does not F, if it is an instance of a type that characteristically Fs, and the type’s characteristically F-ing is a reason for having or maintaining such objects. There is a risk of circularity here, however: it may be that X is only a token of some type if it has a certain function.

61 E Weinrib, *The Idea of Private Law* (OUP 2012).

62 See the claim that ‘necessarily, law is justice-apt’ in L Green, ‘Positivism and the Inseparability of Law and Morals’ (2008) 83 NYU L Rev 1035, 1050. More generally, necessarily, law is apt for assessment in terms of reasons.

63 Weinrib (n 61). Another possibility is that Weinrib’s objection to functionalist explanation is that for F to count as a function of X, X must be capable of *failing* to F. If the supposed function of tort law is said to be to ‘constitute us as moral equals’, then it might be said that tort law cannot *fail* to do this. But this seems untrue: there can be wildly unjust systems of tort law that still count as tort law and may fail to so constitute us. If, however, Weinrib believes that tort law *necessarily* only exists if it constitutes us as moral equals, then this would potentially explain why his account is non-functionalist.

64 We identify the necessary mental element as ‘an intention-like mental state’ rather than straightforwardly as ‘intention’ because the requisite mental state can be of a very low grade, one that does not demand any thoughtfulness, consideration or even consciousness. I can intend to drink the tea sitting in a cup in front of me, and therefore have that as my aim, without much thought, or even subconsciously. While some mental element is essential to having an aim, it is not a particularly demanding element.

65 Elimination of relative disadvantages between social groups may be one such unrealisable aim: T Khaitan, ‘The Point of Discrimination Law’ in M Nussbaum and others (eds), *The Empire of Disgust* (OUP 2018).

## Notes

This work was supported by Oxford-Melbourne Allan Myers Fund.

